# Associations between Neuropsychiatric Symptoms and Alzheimer’s Disease Biomarkers in People with Mild Cognitive Impairment

**DOI:** 10.3390/brainsci13081195

**Published:** 2023-08-12

**Authors:** Giulia Arenare, Riccardo Manca, Paolo Caffarra, Annalena Venneri, on behalf of the Alzheimer’s Disease Neuroimaging Initiative

**Affiliations:** 1 Department of Medicine and Surgery, University of Parma, 43125 Parma, Italy; 2 Department of Life Sciences, Brunel University London, Uxbridge UB8 3BH, UK

**Keywords:** neuropsychiatric symptoms, mild cognitive impairment, biomarkers, amyloid, tau, neurodegeneration

## Abstract

Background: Neuropsychiatric symptoms (NPS) are associated with faster decline in mild cognitive impairment (MCI). This study aimed to investigate the association between NPS severity and Alzheimer’s disease (AD) biomarkers, i.e., amyloid-β (Aβ), phosphorylated tau protein (p-tau) and hippocampal volume ratio (HR), to characterise in more detail MCI patients with a poor prognosis. Methods: A total of 506 individuals with MCI and 99 cognitively unimpaired older adults were selected from the ADNI dataset. The patients were divided into three different groups based on their NPI-Q total scores: no NPS (*n* = 198), mild NPS (*n* = 160) and severe NPS (*n* = 148). Regression models were used to assess the association between the severity of NPS and each biomarker level and positivity status. Results: Cerebrospinal fluid Aβ levels were positively associated with older age and lower MMSE scores, while higher p-tau levels were associated with female sex and lower MMSE scores. Only patients with severe NPS had a lower HR (β = −0.18, *p* = 0.050), i.e., more pronounced medio-temporal atrophy, than those without NPS. Discussion: Only HR was associated with the presence of NPS, partially in line with previous evidence showing that severe NPS may be explained primarily by greater grey matter loss. Future longitudinal studies will be needed to ascertain the relevance of this finding.

## 1. Introduction

Neuropsychiatric symptoms (NPS) represent a prominent clinical manifestation of Alzheimer’s disease (AD). These symptoms may be characterised by disturbed perception (i.e., hallucinations), contents of thoughts (i.e., delusions), mood (i.e., depression, anxiety and apathy) and behaviour (e.g., aggression, disinhibition, appetite problems) and are frequently observed in patients with dementia [1]. These symptoms negatively affect patients’ quality of life, to the extent that caregivers often perceive them as more burdensome than cognitive symptoms [2]. Although fluctuating, the prevalence of NPS increases in parallel with the progression of cognitive decline [3] and with a loss of autonomy in daily life [4]. Indeed, the presence of NPS at an earlier disease stage, such as mild cognitive impairment (MCI), is a negative prognostic indicator and has been associated with faster cognitive decline [5]. 

In line with recent efforts to characterise AD from a biological point of view, the so-called AT(N) framework (i.e., amyloid, tau, neurodegeneration) [6], the relationship between the presence of NPS and detected levels of multiple AD biomarkers has been studied. Investigations have focussed on amyloid beta (Aβ) and phosphorylated tau (p-tau) protein levels as measures of the A and T variables of the AT(N) framework. In fact, the abnormal accumulation of both proteins is considered the primary pathological mechanism of AD. The cerebral accumulation of both Aβ and p-tau has been studied by means of positron emission tomography (PET), while cerebrospinal fluid (CSF) and blood examinations have been used to detect these pathological features as fluid biomarkers. Moreover, total tau (t-tau) and hippocampal volume (HV), assessed via magnetic resonance imaging (MRI), have also been used as indices of neurodegeneration, the N component of the above framework. 

Thus far, heterogeneous results have been reported in relation to the association between different NPS and AD biomarkers. A recent review highlighted that mood symptoms (i.e., depression and anxiety) are primarily associated with higher levels of Aβ, but not of p-tau, in patients with AD [7]. The severity of NPS in AD was also associated with measures of neurodegeneration, e.g., the CSF levels of t-tau. However, a recent study found that affective symptoms predicted progression from MCI to dementia due to AD but were not associated with AD biomarkers [8]. Apathy, the most common NPS in patients with AD [9], has also been found significantly associated with lower CSF levels of Aβ_42_ and reduced HV but not with CSF levels of either t-tau or p-tau [10]. A systematic review focussing on CSF biomarkers, conducted by Showraki et al. [11], showed that the presence of agitation/aggression was consistently associated with higher t-tau and p-tau and lower Aβ_42_ levels. 

Investigations into the severity of NPS seem to suggest that more severe NPS are also primarily associated with greater cerebral Aβ deposition (but not with p-tau load) and with cognitive decline in cognitively unimpaired people [7,12]. Consistently, plasma levels of Aβ_42_/Aβ_40_ were predictive of the severity of mild behavioural impairment (MBI) in people with and without MCI [13]. However, other studies have also shown that more severe NPS correlated, selectively, either with a greater degree of cerebral p-tau load [14] or with worse neurodegeneration [15] across the AD spectrum.

Such inconsistencies in the currently available findings may be due to several reasons. First, NPS are difficult to assess accurately because the scales designed to capture these symptoms differ in the following aspects: (1) the measured construct (e.g., only one symptom vs. several NPS domains), (2) the measurement approach (e.g., clinician-, caregiver- or patient-self-reported) and (3) the rating approach (e.g., frequency, severity, etc.) [16]. Additionally, a positive finding or lack of findings about the association between biomarkers and clinical variables may depend on the different methods of assessment of AD biomarkers (i.e., PET, MRI, CSF and blood) and their level of accuracy. Indeed, different methods may be more appropriate to capture different biological alterations (e.g., Aβ before p-tau accumulation) across the various stages of the AD continuum [17]. As a result, the temporal association between the emergence of NPS, their severity and AD biological changes remains unclear. Clarifying such a relationship in different disease stages may have relevance for the clinical management of people with AD. First, understanding the pathological underpinnings of NPS may aid in the early diagnosis of AD, since a sub-sample of people with AD manifests NPS before cognitive deficits (e.g., those with MBI, [18]). Second, since people with MCI and NPS have a worse prognosis than those without, the timely detection of those characterised by worse AD pathological changes may help clinicians to provide early and targeted treatments in order to lower the risk of progression from MCI to dementia [19]. Third, this knowledge may provide useful insights in the interpretation of the findings of clinical trials targeting NPS and clarify specific mechanisms of action (e.g., either Aβ or p-tau clearance).

Considering the lack of consistency in the currently available findings on the association between NPS and AD biomarkers [20] in people with MCI and NPS, who may have the greatest risk of progression to dementia, the aim of this study was to qualify the association between the most commonly used AD biomarkers (i.e., Aβ and p-tau and hippocampal neurodegeneration) and NPS severity levels in a sample of people with MCI. 

## 2. Material and methods

### 2.1. Study Design and Participants

For this study, 506 patients with MCI were selected from the Alzheimer’s Disease Neuroimaging Initiative (ADNI) database www.adni-info.org (accessed on 31 May 2023), using the following inclusion criteria: (1) a clinical diagnosis of MCI (i.e., a history of cognitive complaint, evidence of cognitive deficits without dementia and preserved functional independence); (2) the availability of data on NPS measured by means of the Neuropsychiatric Inventory Questionnaire (NPI-Q); (3) the availability of data on global cognitive functioning measured by means of the Mini Mental State Examination (MMSE) total score (≥24); and (4) the availability of data on the CSF levels of AD biomarkers (i.e., Aβ, t-tau and p-tau) and a T1-weighted magnetic resonance imaging (MRI) scan.

An additional sample of 99 older adults with CSF biomarkers of AD within the normal range and no evidence of cognitive impairments at any of the time-points available in the ADNI database were also selected to characterize the clinical and biomarker profiles of patients.

The ADNI protocol (for full details, see: https://adni.loni.usc.edu/methods/documents/ (accessed on 10 July 2023)) received ethical approval from the institutional review board at each centre of recruitment and all participants provided informed consent. Secondary analyses were carried out for this study in compliance with the Declaration of Helsinki, and ethical approval was granted by the Research Committee of Brunel University London (reference number 30422-TISS-Jul/2021-33453-2).

The ADNI was launched in 2003 as a public–private partnership, led by Principal Investigator Michael W. Weiner, MD. The primary goal of the ADNI is to test whether serial magnetic resonance imaging (MRI), positron emission tomography (PET), other biological markers and clinical and neuropsychological assessment can be combined to measure the progression of mild cognitive impairment (MCI) and early Alzheimer’s disease (AD). For up-to-date information, see www.adni-info.org.

### 2.2. NPS Assessment

The NPI-Q is a questionnaire designed to be completed by informants to provide information about a list of 12 NPS observed in the patients they care for. The total NPI-Q score is calculated as the sum of the severity scores (0 if a symptom is absent, from 1 to 3 for symptoms that are present) of all the individual symptoms and ranges from 0 to 36 [21].

The patient sample was divided into 3 different groups based on their NPI-Q total scores: an absence of NPS (NPI-Q = 0), low NPS severity (NPI-Q ≤ 2) and high NPS severity (NPI-Q > 2). The NPI-Q value of 2, used to stratify participants with low and high NPS severity, was the median score of the sub-group of patients presenting symptoms after removing those with no NPS.

### 2.3. CSF-Derived Biomarkers for Aβ and p-tau

Fluid biomarkers for Aβ and p-tau pathological alterations associated with AD were used in this study. The details on CSF biomarker data collection as part of the ADNI have been published previously [22,23]. Participants were considered to be amyloid-positive if their CSF Aβ_1–42_ level was equal to or below 977 pg/mL [24], while positivity for the p-tau biomarker was determined if their CSF p-tau level was equal to or above the cut-off of 26.64 [25]. 

### 2.4. MRI-Derived Biomarker for Neurodegeneration

Details on the protocols for MRI data acquisition used for the ADNI are available at http://adni.loni.usc.edu/methods (accessed on 10 July 2023). A standard voxel-based morphometry pre-processing pipeline was implemented using the Statistical Parametric Mapping (SPM) 12 software (Wellcome Centre for Human Neuroimaging, London, UK), running on MATLAB R2014a, version 8.3 (The MathWorks, Inc., Natick, MA, USA). Structural T1-weighted MRI scans were reoriented to the anterior commissure–posterior commissure line and segmented into grey matter (GM), white matter (WM) and cerebrospinal fluid (CSF) maps. Total GM, WM, and CSF volumes were determined using maps saved in their native space by means of the ‘get_totals’ script (http://www0.cs.ucl.ac.uk/staff/g.ridgway/vbm/get_totals.m (accessed on 10 July 2023)). Subsequently, total intracranial volume (TIV) was calculated by summing the GM, WM and CSF total volumes for each participant.

HV was calculated by creating a mask of the bilateral hippocampi with the WFU PickAtlas toolbox and then using the “get_totals” script to extract the total HV from each previously segmented GM map. Finally, the hippocampal ratio (HR) was calculated by dividing the resulting HV by the TIV, and this parameter was used as a biomarker for neurodegeneration [6]. In detail, patients were considered positive for this biomarker if their HR was at least 2 standard deviations below the mean HR value of the healthy control group.

### 2.5. Statistical Analysis

All demographic and clinical variables and CSF biomarker levels were compared between the MCI and cognitively unimpaired groups using an independent-sample *t*-test for normally distributed variables and a Mann–Whitney *U* test for those not normally distributed. The *chi-square* test was used to test for sex, binary biomarker positivity variables and NPS severity group. After stratifying the patient groups according to NPS severity, the demographic and clinical variables were compared between groups using ANOVA, while the *chi-square* test was used for sex.

Since the cut-offs for AD biomarkers are arbitrary and not universally defined, in this study, two complementary approaches were used: (1) linear multiple regression models were used to assess the association between the severity of NPS and each CSF biomarker level in the patient sample, with total MMSE score, age, sex and education included as covariates; (2) logistic regression models were used to determine the association between the severity of NPS and biomarker positivity status in the patient sample, with total MMSE score, age, sex and education included as covariates.

A two-tailed significance threshold value of *p* < 0.05 was used for all models. The RStudio software (Version 1.3.1056, Boston, MA, USA) was used for all statistical analyses.

## 3. Results

### 3.1. Demographic and Cognitive Data of Patients and Controls

No significant differences were found between the patients and controls for any of the socio-demographic variables, while the patient group had an MMSE score significantly lower than that of the cognitively unimpaired group. The demographic and clinical features of the patients and controls are summarised in Table 1. 

As expected, the patients presented with higher NPI-Q total scores than the control group (*t*(603) = −5.44, *p* < 001). Moreover, their CSF levels of p-tau were significantly higher, while the CSF levels of Aβ and HR values were significantly lower in the patient sample when compared with the controls. Consistently, the proportion of patients who were identified as positive for each biomarker and with either mild or severe NPS was significantly higher than that among controls.

The groups of patients stratified according to the severity of NPS were comparable in terms of age and education and their MMSE scores (Table 2). However, the group with severe NPS had a higher proportion of males than the other groups. The group with severe NPS had higher rates of all NPS than that with mild NPS, except for hallucinations and aberrant motor behaviours (Supplementary Table S1). However, these individuals showed substantially overlapping behavioural deficits, with irritability and depression among the most frequently present symptoms.

### 3.2. Association between Biomarker Values and NPS 

The results of the multiple regression models showed that a lower HR was significantly predicted by older age and a higher MMSE score (Table 3). Moreover, patients with severe but not mild NPS had a significantly lower HR than those without NPS. 

Aβ levels were significantly associated with older age and lower MMSE scores, while lower p-tau levels were significantly associated with male sex and higher MMSE scores. However, neither Aβ nor p-tau CSF levels were associated with the presence of NPS, irrespective of their severity.

### 3.3. Association between Biomarker Positivity and NPS

The logistic regression analyses revealed that older age and a lower MMSE score were significantly associated with positivity status for all biomarkers, while male sex was associated with a lower likelihood of p-tau positivity (Table 4). Consistent with the linear regression analyses, the group with severe NPS alone, but not that with mild NPS, had a higher likelihood of neurodegeneration biomarker positivity (i.e., a HR above the cut-off value) than the patient group without NPS. In contrast, the severity of NPS was not associated with positivity status for any other biomarkers.

## 4. Discussion

This study investigated the association between AD biomarkers and the severity of NPS in patients experiencing MCI due to AD, focussing on NPS that negatively impact on patients’ and caregivers’ quality of life. Among the three biomarkers investigated (i.e., CSF Aβ and p-tau levels and HR), only HR was negatively associated with NPS severity, while Aβ and p-tau were primarily associated with demographic characteristics.

The lack of a significant association between Aβ and NPS severity is partially in line with previous studies. In fact, a comprehensive systematic review by Ng et al. [7] showed that the severity of mood disorders and CSF Aβ levels were not consistently associated in cross-sectional investigations. Another study using the ADNI database also found that CSF-based AD biomarkers did not differ between three groups of people with MCI with either affective, agitation/aggression or no NPS [8]. In contrast, other studies have found a significant association between lower CSF levels of Aβ and both the presence and greater severity of specific NPS, such as depression, anxiety and apathy [10,26]. However, it must be mentioned that this relationship appeared to be mediated by the severity of cognitive decline (i.e., MMSE score) [10]. This finding might have different implications. It is possible that associations between the severity of Aβ pathology, assessed using CSF, and NPS may be detected primarily in people with more severe cognitive deficits. Therefore, failing to control for the degree of cognitive decline may lead to biased results. Consistent with this interpretation, in this study, MMSE scores were significantly associated with worse biomarker profiles, i.e., lower Aβ and higher p-tau CSF levels and a smaller HR. Additionally, NPS–biomarker associations may manifest when heterogeneous samples characterised by a wide range of AD pathologic severity are investigated. However, the sample selected for this study included patients with MCI alone, who were possibly characterised by mild AD pathological changes. Indeed, only two in three people with MCI were positive for Aβ, and this might have prevented the detection of significant associations. 

Similarly, no association was found between the CSF levels of p-tau and the probability of presenting with NPS, irrespective of symptom severity. Previous studies focussing on the severity of depressive symptoms in people both with [8,27] and without MCI [28] found no significant associations with CSF levels of p-tau. Only Krell-Roesch et al. [26] found that a higher p-tau/Aβ_42_ ratio was associated with more severe depression and anxiety in a sample of cognitively unimpaired older adults and patients with MCI. The consistent lack of significant associations between NPS and p-tau may be due to the fact that p-tau changes occur later in the disease course, compared with Aβ accumulation, and are associated with cognitive decline [29]. Hence, it is possible that p-tau levels may be more strongly associated with NPS severity in later disease stages. Indeed, a study found no significant association between p-tau and NPS in patients with MCI, while an association was detected in patients with dementia [30]. In line with this hypothesis, only 45% of the patients included in that study were positive for p-tau, thus suggesting that this sample presented with mild biomarker alterations. 

Finally, the presence of severe NPS but not mild NPS was significantly associated with greater levels of neurodegeneration when patients were compared with those who presented no NPS. This was the case independently of whether HR was treated as either a continuous or a binary variable. Various neuroimaging studies that have attempted to detect neuroimaging biomarkers of NPS across the AD continuum have shown associations between more severe NPS and both GM and WM damage [7,31]. In particular, one study found a significant association between a composite MRI-based index of neurodegeneration (i.e., a combination of hippocampal volume and the cortical thickness of brain regions typically affected by AD pathology) and different clusters of NPS [32]. The volumetric and cortical thickness values of various medial temporal structures have been found to be significant predictors of NPS and MBI in people with and without MCI [33,34]. Consistent evidence has also emerged from studies investigating CSF biomarkers of neurodegeneration, e.g., t-tau/Aβ_42_ [15,26]. Therefore, the findings of this study and previous investigations seem to suggest that people with MCI and severe NPS may show signs of more advanced AD-related neurodegeneration than patients with no NPS, even though their global cognitive status appears comparable.

It is worth noting that several of the demographic variables we controlled for were associated with different AD biomarkers. In particular, age was associated with generally worse biomarker profiles, i.e., higher p-tau and lower Aβ and HR values. More years in education were positively associated with HR, thus suggesting a protective influence of education on the integrity of the hippocampus even in the presence of AD pathology. Finally, the male sex was found to be associated with lower p-tau CSF levels and a lower likelihood of being p-tau positive, thus suggesting a less severe neuropathological profile in male participants. A greater AD neuropathological burden in women has already been reported in the early and preclinical stages of AD [35]. In contrast, a higher proportion of male participants was observed in the group with severe NPS than in the other groups, a finding that is not consistent with previous evidence showing that the female sex may be associated with a greater risk of more severe NPS, except for apathy [36]. Such an observation may be due to unexplored prior psychiatric diagnoses but also a multitude of unknown sex- and gender-related psychosocial, genetic and neuropathological factors. However, it must be mentioned that the number of male and female participants included in this study was unbalanced and potentially not representative of this clinical population.

This study has limitations. First, the NPI-Q is a questionnaire based on responses provided, in most cases, by caregivers and therefore represents a subjective and potentially biased evaluation of the behavioural profiles of patients. Some of the questions included in the NPI-Q may be challenging and stressful for people who care for patients with cognitive impairments, especially relatives who have daily contact with them, and this could influence their evaluation. An in-depth interview performed by a trained clinician with patients and caregivers would be ideal for ascertaining the presence and severity of NPS. Nevertheless, it must be mentioned that the NPI-Q is easy to administer and has been used extensively to investigate behavioural problems experienced by people with dementia. This is an advantage that enables more direct comparisons with other similar studies. Second, the sample of participants with MCI included in this study presented primarily with either no or mild NPS. Moreover, it included a majority of male participants, different from the populations usually encountered in real-life clinical settings. This may be the outcome of the participant selection process based on the availability of a range of clinical and biological data in the ADNI database. Therefore, it is not possible to rule out completely that such a selection might have limited the sample size and introduced a bias in our sample. As a consequence, these factors may limit the generalisability of these findings, especially because men and women may show different degrees of neuropathological changes in the early stages of disease [35]. Fourth, we assessed NPS severity globally using the NPI-Q total score, and this prevents any definite conclusions on the possible differential associations between AD biomarkers and individual NPS [26]. In fact, this study focussed on the severity of NPS globally, while previous studies detected differential, although heterogeneous, associations with specific NPS [10,26]. Fifth, this study used a cross-sectional design with a patient sample only including people in the early clinical stages of AD, i.e., MCI. Thus, it is only possible to speculate on how the relationship between AD biomarkers and NPS might differ across the AD continuum and on the relationship between longitudinal changes in biomarkers and symptoms as the disease progresses. Indeed, future studies will be needed to clarify whether accumulation of AD-related neuropathological changes (e.g., Aβ) may determine the severity and type of NPS profiles in cognitively unimpaired older adults before a substantial degree of neurodegeneration has occurred [14], although the evidence accumulated to date appears to still be inconsistent [7].

## 5. Conclusions

This study found that the presence of severe NPS in patients with MCI may be explained primarily by greater levels of AD-related GM neurodegeneration (i.e., lower HR values). This finding suggests that the early detection of NPS may be beneficial in preventing a more severe neuropathological trajectory. Indeed, targeting patients with MCI and NPS with either pharmacological or non-pharmacological treatments that could support brain plasticity and slow AD-related neurodegenerative processes may delay functional decline and improve prognosis. 

Longitudinal investigations into the association between different biomarkers of AD and specific NPS would be needed to ascertain possible specific vulnerabilities in sub-groups of patients who may be at increased risk of progression from MCI to AD dementia. Establishing patterns of biomarker–NPS relationships across the AD continuum would offer a biological model with which to interpret symptomatic changes occurring both naturally and as a result of targeted interventions. Future research would benefit from a comparison of the association between different AD biomarkers and individual NPS, as well as the severity and progression of symptoms. In particular, CSF- and blood-based levels of Aβ_40_, Aβ_42_, Aβ_42_/Aβ_40_, p-tau_181_, p-tau_217_ and p-tau_231_ would be ideal candidates for assessing the involvement of Aβ and tau pathological changes. Moreover, neurofilament light chain (NfL) could be investigated as a marker of neurodegeneration, since people with MCI and MBI appear to show greater NfL increase in plasma over a period of 2 years [37]. Indeed, MBI criteria have been devised to detect NPS with an onset in older adulthood that are persistent and not explained by other medical conditions [16]. MBI is considered to be an “at-risk state” that could possibly capture those people who are more likely to progress to MCI and dementia [38]. Fluctuating NPS and a variety of other factors, including prior psychiatric history, may influence their manifestation. Therefore, focussing more efforts on clarifying the biomarker profile of MBI in different disease stages may lead to clinically relevant insights to support both the early diagnosis and clinical management of people with early AD.

## Figures and Tables

**Table 1:** Demographic, clinical and biomarker profiles of patients and controls. Values are means ± standard deviations, unless otherwise specified.

Variable	Patients (*n* = 506)	Controls (*n* = 99)	t	*p*
Sex (M/F, %) ^a^	59.7/40.3%	55.6/44.4%	0.58 ^b^	0.445
Age	72.33 ± 7.42	72.22 ± 5.05	−0.13	0.893
Education ^c^	16.00 (4.00)	16.00 (3.00)	0.63 ^d^	0.531
MMSE ^c^	28.00 (2.00)	29.00 (1.00)	7.25 ^d^	<0.001
NPI-Q score ^c^	1.00 (3.00)	0.00 (0.00)	−7.21 ^d^	<0.001
Aβ (pg/mL) ^c^	819.90 ± 659.25	1563.00 ± 608.50	10.48 ^d^	<0.001
p-tau (pg/mL) ^c^	24.95 ± 17.84	18.55 ± 5.98	−6.97 ^d^	<0.001
HR (‰)	5.07 ± 0.52	5.39 ± 0.32	5.93	<0.001
NPS group (severe/mild/none) ^a^	29.2%/31.6%/39.1%	6.1%/15.2%/78.8%	53.80 ^b^	<0.001
Aβ (negative/positive) ^a^	37.0%/63.0%	100.0%/0.0%	132.03 ^b^	<0.001
p-tau (negative/positive) ^a^	54.7%/45.3%	100.0%/0.0%	72.09 ^b^	<0.001
HR (negative/positive) ^a^	77.1%/22.9%	98.0%/2.0%	23.05 ^b^	<0.001

Aβ: Amyloid beta, F: Females, HR: Hippocampal ratio, M: Males, MMSE: Mini Mental State Examination, NPI-Q: Neuropsychiatric Inventory Questionnaire, NPS: Neuropsychiatric symptoms, p-tau: Phosphorylated tau; ^a^ Proportions; ^b^ *Chi-square* test; ^c^ Median (interquartile range); ^d^ Mann–Whitney *U* test (standardised).

**Table 2:** Comparisons of clinical and demographic profiles of patients stratified according to neuropsychiatric symptom severity (ANOVAs). Values are means ± standard deviations, unless otherwise specified.

Variable	No NPS (*n* = 198)	Mild NPS (*n* = 160)	Severe NPS (*n* = 148)	*F*	*p*
Sex (M/F, %) ^a^	54.5/45.5%	55.6/44.4%	70.9/29.1%	11.07 ^b^	0.004
Age	72.58 ± 7.44	71.73 ± 7.51	72.64 ± 7.29	0.76	0.471
Education	16.17 ± 2.91	16.31 ± 2.64	16.12 ± 2.72	0.20	0.816
MMSE	27.75 ± 1.75	27.86 ± 1.88	27.81 ± 1.70	0.16	0.850

F: Females, M: Males, MMSE: Mini Mental State Examination, NPS: Neuropsychiatric symptoms; ^a^ Proportions; ^b^ *Chi-square* test.

**Table 3:** Results of the multiple regression model for biological factors. Values are standardised coefficients (95% confidence interval).

Variables	Biomarkers		
	Aβ	p-tau	HR
Sex (M)	−0.16 (−0.34, 0.02), *p* = 0.076	−0.29 (−0.47, −0.11), *p* = 0.002	0.1550 (−0.31, 0.01) *p* = 0.070
Age	−0.13 (−0.21, −0.04), *p* = 0.003	0.07 (−0.02, 0.16), *p* = 0.124	−0.42 (−0.50, −0.34), *p* < 0.001
Education	0.01 (−0.08, 0.09), *p* = 0.858	−0.03 (−0.12, 0.05), *p* = 0.460	−0.09 (−0.16, −0.02), *p* = 0.019
MMSE	0.20 (0.11, 0.28), *p* < 0.001	−0.21 (−0.92, −0.12), *p* < 0.001	0.19 (0.12, 0.27), *p* < 0.001
NPS group:			
*Mild—No NPS*	−0.16 (−0.36, 0.04), *p* = 0.118	−0.02 (−0.21, 0.19), *p* = 0.881	0.04 (−0.14, 0.22), *p* = 0.657
*Severe—No NPS*	−0.16 (−0.37, 0.05), *p* = 0.127	0.20 (<−0.01, 0.41), *p* = 0.061	−0.18 (−0.37, <−0.01), *p* = 0.050

Aβ: Amyloid beta, HR: Hippocampal ratio, M: Male, MMSE: Mini Mental State Examination, NPS: Neuropsychiatric symptoms, p-tau: Phosphorylated tau.

**Table 4:** Results of logistic regression analyses for the positivity of biological factors. Values are odds ratios (95% confidence interval).

Variables	Biomarkers		
	Aβ (Positive)	p-tau (Positive)	HR (Positive)
Sex (M)	1.32 (0.89, 1.96), *p* = 0.174	0.66 (0.45, 0.98), *p* = 0.039	1.33 (0.81, 2.20) *p* = 0.263
Age	1.04 (1.01, 1.07), *p* = 0.006	1.03 (1.00, 1.05), *p* = 0.037	1.12 (1.08, 1.16), *p* < 0.001
Education	0.98 (0.91, 1.05), *p* = 0.555	1.01 (0.95, 1.09), *p* = 0.660	1.04 (0.96, 1.14), *p* = 0.306
MMSE	0.80 (0.71, 0.89), *p* < 0.001	0.77 (0.69, 0.86), *p* < 0.001	0.81 (0.71, 0.92), *p* = 0.002
NPS group:			
*Mild—No NPS*	1.13 (0.72, 1.77), *p* = 0.587	1.02 (0.66, 1.58), *p* = 0.937	0.91 (0.52, 1.61), *p* = 0.752
*Severe—No NPS*	1.11 (0.70, 1.76), *p* = 0.659	1.37 (0.88, 2.14), *p* = 0.170	1.85 (1.08, 3.17), *p* = 0.024

Aβ: Amyloid beta, HR: Hippocampal ratio, M: Male, MMSE: Mini Mental State Examination, NPS: Neuropsychiatric symptoms, p-tau: Phosphorylated tau.

## Data Availability

All ADNI data are made publicly available upon request.

## Supplementary Materials

The following supporting information can be downloaded at: https://www.mdpi.com/article/10.3390/brainsci13081195/s1, Table S1. Differences in the prevalence of individual neuropsychiatric symptoms between patient groups (*Chi-square* test). All values are frequencies (proportions).
