# Supplementary material for: Associations between Neuropsychiatric Symptoms and Alzheimer’s Disease Biomarkers in People with Mild Cognitive Impairment
Source: Brain Sci. 2023 Aug 12;13(8):1195. doi: 10.3390/brainsci13081195 (PMC10452057; doi:10.3390/brainsci13081195)
Supplement: Supplementary file 1 [file brainsci-13-01195-s001.zip › brainsci-2527515-supplementary.pdf]

**Table S1.** Differences in the prevalence of individual neuropsychiatric symptoms between patient groups (*Chi-square* test). All values are frequencies (proportions).

| **Symptom** | **No NPS (*n* = 198)** | **Mild NPS (*n* = 160)** | **Severe NPS (*n* = 148)** | $\chi^2$ | ***p*** |
|---|---|---|---|---|---|
| Delusions | 0 (0.0%) | **0 (0.0%)** | **7 (4.7%)** | -[a] | <0.001 |
| Hallucinations | 0 (0.0%) | 0 (0.0%) | 3 (2.0%) | -[a] | 0.025 |
| Agitation | 0 (0.0%) | **28 (17.5%)** | **58 (39.5%)** | 92.24 | <0.001 |
| Depression | 0 (0.0%) | **43 (26.9%)** | **83 (56.1%)** | 142.92 | <0.001 |
| Anxiety | 0 (0.0%) | **16 (10.0%)** | **58 (39.5%)** | 109.07 | <0.001 |
| Euphoria | 0 (0.0%) | **0 (0.0%)** | **13 (8.8%)** | -[a] | <0.001 |
| Apathy | 0 (0.0%) | **14 (8.8%)** | **64 (43.2%)** | 129.44 | <0.001 |
| Disinhibition | 0 (0.0%) | **4 (2.5%)** | **43 (29.1%)** | 97.65 | <0.001 |
| Irritability | 0 (0.0%) | **44 (27.5%)** | **93 (63.3%)** | 179.83 | <0.001 |
| Aberrant motor behaviours | 0 (0.0%) | 8 (5.0%) | 14 (9.5%) | 18.60 | <0.001 |
| Night-time behaviours | 0 (0.0%) | **38 (31.1%)** | **60 (40.5%)** | 91.63 | <0.001 |
| Appetite | 0 (0.0%) | **8 (5.0%)** | **39 (26.4%)** | 92.49 | <0.001 |

NPS: Neuropsychiatric symptoms

[a] *Fisher's Exact* Test

In bold: rates significantly different between patient groups with mild and severe neuropsychiatric symptoms
